# Dataset for the thermodynamic description for the NaF-KF-RbF-ZnF_2_ system

**DOI:** 10.1016/j.dib.2018.10.087

**Published:** 2018-11-05

**Authors:** Huiqin Yin, Shuang Wu, Xueliang Wang, Long Yan, Wenguan Liu, Zhongfeng Tang

**Affiliations:** Shanghai Institute of Applied Physics, Chinese Academy of Sciences, Shanghai 201800, PR China

## Abstract

This data article includes the supplementary database material for the manuscript “Thermodynamic description for the NaF-KF-RbF-ZnF_2_ system”, (Tang et al., 2018). We present all the thermodynamic parameters used and optimized model parameters of the thermodynamic database of the NaF-KF-RbF-ZnF_2_ quaternary system, by which researchers can obtain the calculated phase diagrams. What׳s more, the comparison of calculations with experimental lattice constants of all compounds and relative deviations of unit cell parameters between before and after structure optimization for the NaF-KF-RbF-ZnF_2_ system are demonstrated in this work. Meanwhile, we provide the calculated mixing enthalpy in the binary AF-ZnF_2_ (A=Na, K and Rb) and AF-RbF (A=Na and K) system, which are helpful for researchers to better understand the system. These supplementary databases are most useful in understanding the manuscript (Tang et al., 2018).

**Specifications table**

**Table:** 

Subject area	*Chemistry*
More specific subject area	*Physical Chemistry, Thermodynamics*
Type of data	*Tables and figures*
How data was acquired	*Researcher made questionnaire analysis*
Data format	*Raw and analyzed*
Experimental factors	*Lattice constants of all compounds were conducted from first-principles calculation, and mixing enthalpy in liquid phase was plotted through the PanPhaseDiagram module of PANDAT software.*
Experimental features	*The mixing enthalpy in liquid phase was carried out in two steps: the Gibbs free energy of liquid phase was calculated firstly, then, the mixing enthalpy in liquid phase can be calculated on the basis of the thermochemistry value.*
Data source location	*Shanghai, China (Shanghai Institute of Applied Physics, Chinese Academy of Sciences; Jialuo Road 2019, Jiading District)*
Data accessibility	*All data are presented in this article*
Related research article	Huiqin Yin, Shuang Wu, Xueliang Wang, Long Yan, Wenguan Liu, Zhongfeng Tang, Thermodynamic description for the NaF-KF-RbF-ZnF_2_ system, Journal of Fluorine Chemistry, (2018) in press. [1]

**Value of the data**

• The data list all the thermodynamic parameters used and optimized model parameters of the thermodynamic database of the NaF-KF-RbF-ZnF_2_ quaternary system, which is useful for investigators to calculate the relevant phase diagrams.
• The data show the comparison of calculated with experimental lattice constants of all compounds and relative deviations of unit cell parameters between before and after structure optimization for the NaF-KF-RbF-ZnF_2_ system. These values are useful to understand the lattice constants of all compounds in this system.
• The data present the calculated mixing enthalpy in the binary AF-ZnF_2_ (A=Na, K and Rb) and AF-RbF (A=Na and K) system, which are helpful for researchers to better understand the system.

## 1 Data

Table 1, Table 2 list all the thermodynamic parameters used and optimized model parameters of the thermodynamic database of the NaF-KF-RbF-ZnF_2_ quaternary system.

**Table 1:** Thermodynamic parameters for the NaF-KF-RbF-ZnF_2_ system.

**Compound**	**Gibbs Energy/J**	**Temp./K**
NaF	−592757.502 + 307.110402*T-53.29914*T*LN(T) + 0.003451047*T**2 – 2.26795167E-6*T**3 + 248,839.2*T**(−1)	298–1269
	−594935.992 + 393.386907*T−64.62899*T*ln(T) + 2.769787E−4*T**2−1.64256167E−8*T**3 – 4895866*T**(−1)	1269–3500
NaF (liquid)	+ GNaF + 33,346.5−26.2777778*T	298–3000
KF	−583869.251 + 257.421863*T-47.79132*T*ln(T) − 0.0046140735*T**2 – 6.56616e-007*T**3 + 84,928.5*T**(−1)	298–900
	−566044.319 + 135.998567*T − 32.01124*T*ln(T)-0.005759505*T**2-1.993335e−006*T**3 – 2839890*T**(−1)	900–1131
	−603118.731 + 438.402642*T−71.9648*T*ln(T)	1131–3000
KF(liquid)	+ GKF + 27196-24.045977*T	298– 3000
ZnF_2_	−767801.199904635 + 767.418160486415*T−111.3*T*LN(T)−127200*T**(-1)-3352.8*T**(0.5)	298–600
	−784693.845952652 + 354.466306900808*T−62.59*T*LN(T)−0.012865*T**2	600–1090
	−784693.845952652 + 354.466306900808*T−62.59*T*ln(T)−0.012865*T**2	1019–1220
ZnF_2_(liquid)	−727469.612944635 + 732.384921069322*T−111.3*T*LN(T)−127200*T**(−1)-3352.8*T**(0.5)	298–600
	−744362.258992652 + 319.433067483715*T−62.59*T*LN(T)−0.012865*T**2	600–1000
	−769323.258992652 + 592.821818666493*T−100.416*T*ln(T)	1000–1776
RbF	−563090.816619278 + 158.255830896839*T−33.329744*T*LN(T)−0.01926732*T**2 – 251040*T**(−1)	298–1500
	−558571.050619278 + 313.921385717032*T−58.9944*T*LN(T)	1500–1663
RbF(liquid)	−546097.479600841 + 300.480210863334*T-58.9944*T*LN(T)	298–1066
	−297259.229074478-607.416841145761*T + 47.291752*T*LN(T)−0.001847236*T**2 – 73360164*T**(−1)	1066–1200
	−544409.531634478 + 298.957487046549*T−58.9944*T*LN(T)	1200–1663

**Table 2:** All the optimized model parameters of the NaF-KF-RbF-ZnF_2_ system (*: This work).

**NaF-ZnF**_**2**_**system**	**Resource**	**NaF-RbF system**	**Resource**
L0NaF,ZnF2Liquid=-81235.6+35T	[*]	L0NaF,RbFLiquid=-8043.9+20T	[*]
L1NaF,ZnF2Liquid=-36286.4+30.22T	[*]	L1NaF,ZnF2Liquid=-6013.33-7.23T	[*]
$G_{NaZnF_{3}}^{Solid}=G_{NaF}^{Solid}+G_{ZnF_{2}}^{Solid}-15040.1-8.2T$	[*]	L2NaF,ZnF2Liquid=-888.51+1.61T	[*]
**RbF-ZnF**_**2**_**system**		**KF-ZnF**_**2**_**system**	
L0RbF,ZnF2Liquid=-45526.9-26.69T	[*]	L0KF,ZnF2Liquid=-74088.2+28.20T	[*]
L1RbF,ZnF2Liquid=-18941+29.44T	[*]	L1KF,ZnF2Liquid=-20749.8+13.20T	[*]
$G_{RbZnF_{3}}^{Solid}=G_{RbF}^{Solid}+G_{ZnF_{2}}^{Solid}-40570.3$	[*]	$G_{KZnF_{3}}^{Solid}=G_{KF}^{Solid}+G_{ZnF_{2}}^{Solid}-33070.78$	[*]
$G_{K_{2}ZnF_{4}}^{Solid}=2\times G_{KF}^{Solid}+G_{ZnF_{2}}^{Solid}-46542.89$	[*]	$G_{K_{2}ZnF_{4}}^{Solid}=2\times G_{KF}^{Solid}+G_{ZnF_{2}}^{Solid}-36812.2$	[*]
**KF-RbF system**			
L0KF,RbFLiquid=-6085.78+0.9T	[*]		
L0KF,RbFSolid=-1746.29+1.04T	[*]		

Table 3, Table 4 present the comparison of calculated with experimental lattice constants of all compounds and relative deviations of unit cell parameters between before and after structure optimization for the NaF-KF-RbF-ZnF_2_ system (experimental data in Parentheses). As observed in Table 4, the maximal relative deviations of unit cell lattice constants before and after the lattice relaxation are less than 4.7%, and the largest deviation of the largest volumes are less than 14.6%. The calculated parameters are reasonable and acceptable, considering allowable experimental errors introduced in this paper and the enthalpy of formation independent of temperature.

**Table 3:** Comparison of calculated with experimental lattice constants of all compounds included in the NaF-KF-RbF-ZnF2 system (experimental data in Parentheses).

**compound**	**Sapce group**		**Lattice parameters (Å)**			**Volume (Å3 cell-1)**
			**a**	**b**	**C**	
RbF	$Pm\overset{¯}{3}m$	3.421(3.27)		3.421(3.27)	3.421(3.27)	40.044(34.97)
RbF	$Fm\overset{¯}{3}m$	5.724(5.64)		5.724(5.64)	5.724(5.64)	187.502(179.41)
RbF	$Fm\overset{¯}{3}m$	3.425(3.29)		3.425(3.29)	3.425(3.29)	40.192(35.61)
ZnF_2_	*P*4_2_/*mnm*	4.788(4.7048)		4.788(4.7048)	3.184(3.1338)	72.986(69.37)
ZnF_2_	*Pbcn*	4.752(4.683)		5.761(5.658)	5.240(5.166)	143.441(136.88)
NaZnF_3_	*Pnma*	5.647(5.5928)		7.846(7.7747)	5.444(5.4186)	241.171(235.61)
NaZnF_3_	*Pbnm*	5.438(5.409)		5.645(5.579)	7.846(7.765)	240.849(234.32)
RbZnF_3_	*P*6_3_/*mnc*	6.001(5.9)		6.001(5.9)	14.679(14.433)	457.776(435.1)
K_2_ZnF_4_	*I*4/*mmm*	4.128(4.058)		4.128(4.058)	13.334(13.109)	227.203(215.87)
KZnF_3_	$Pm\overset{¯}{3}m$	4.129(4.05)		4.129(4.05)	4.129(4.05)	70.375(66.43)

**Table 4:** Relative deviation ($\frac{M_{after-optimization}-M_{before-optimization}}{M_{before-optimization}}$) of unit cell parameters between before and after structure optimization for the NaF-KF-RbF-ZnF_2_ quaternary system.

**compound**	**Sapce group**	**Lattice parameters (Å)**			**Volume (Å3 cell-1)**
		**a**	**b**	**c**	
RbF	$Pm\overset{¯}{3}m$	4.6177%	4.6177%	4.6177%	14.5096%
RbF	$Fm\overset{¯}{3}m$	1.4894%	1.4894%	1.4894%	4.5103%
RbF	$Fm\overset{¯}{3}m$	4.1033%	4.1033%	4.1033%	12.8672%
ZnF_2_	*P*4_2_/*mnm*	1.7684%	1.7684%	1.6019%	5.2126%
ZnF_2_	*Pbcn*	1.4734%	1.8204%	1.4324%	4.7932%
NaZnF_3_	*Pnma*	0.9691%	0.9171%	0.4688%	2.3603%
NaZnF_3_	*Pbnm*	0.5361%	1.1830%	1.0431%	2.7864%
RbZnF_3_	*P*6_3_/*mnc*	1.7119%	1.7119%	1.7044%	5.2117%
K_2_ZnF_4_	*I*4/*mmm*	1.7250%	1.7250%	1.7164%	5.2499%
KZnF_3_	$Pm\overset{¯}{3}m$	1.9506%	1.9506%	1.9506%	5.9386%

Table 5 shows the relative deviation of calculated invariant points for the ternaries from the NaF-KF-RbF-ZnF_2_ quaternary system. It can be seen that the average relative deviation is 17.38%, which is acceptable concerning the errors.

**Table 5:** Relative deviation () of calculated invariant points for the ternaries from the NaF-KF-RbF-ZnF_2_ quaternary system (* and ※ refer to the value from phase diagram calculation and predicted value using the method in this work, respectively).

**Ternary system (A-B-C)**	**Reaction type**	$\chi_{A}$	$\chi_{B}$	$\chi_{C}$	**Resource**
KF-NaF-ZnF_2_	$Liquid\overset{E1}{⟶}K_{2}ZnF_{4}+Halite\#2+Halite\#1$	0.5259	0.2944	0.1797	[*]
		0.622	0.222	0.156	[※]
**Relative deviation (****)**		18.27%	−24.59%	-13.19%	
KF-RbF-ZnF_2_	$Liquid\overset{E2}{⟶}Solid+RbZnF_{3}+Rb_{2}ZnF_{4}$	0.3003	0.4744	0.2253	[*]
		0.263	0.560	0.177	[※]
**Relative deviation (****)**		−12.42%	18.04%	−21.44%	
NaF-RbF-ZnF_2_	Liquid⟶E3NaF+RbZ2nF4+RbF	0.1935	0.6478	0.1587	[*]
		0.130	0.699	0.171	[※]
**Relative deviation (****)**		−32.82%	7.90%	7.75%	

**Fig. 1:**
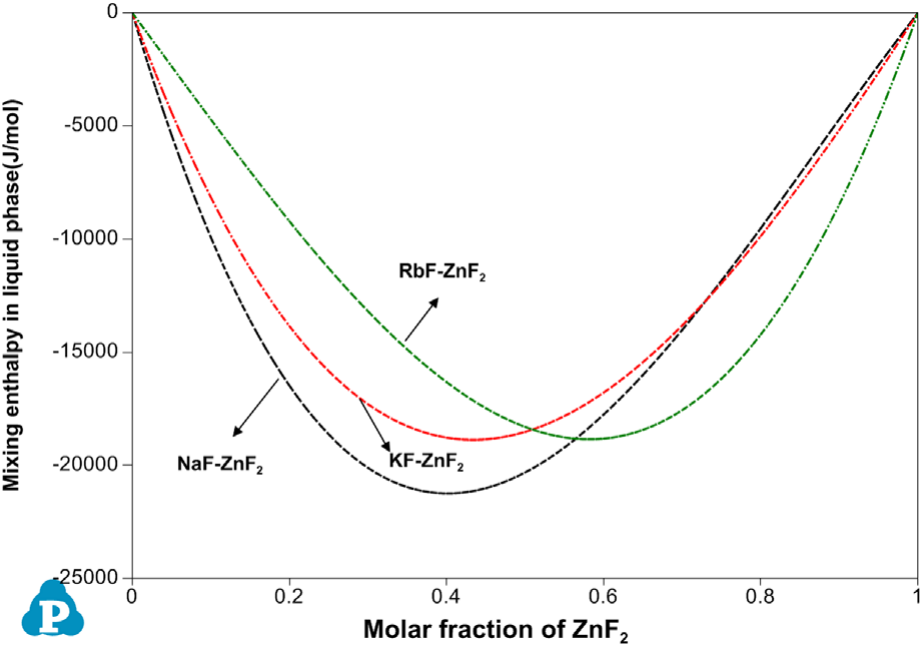
Calculated mixing enthalpy in liquid phase of AF-ZnF_2_ (A=Na, K and Rb) at 1500°C.

**Fig. 2:**
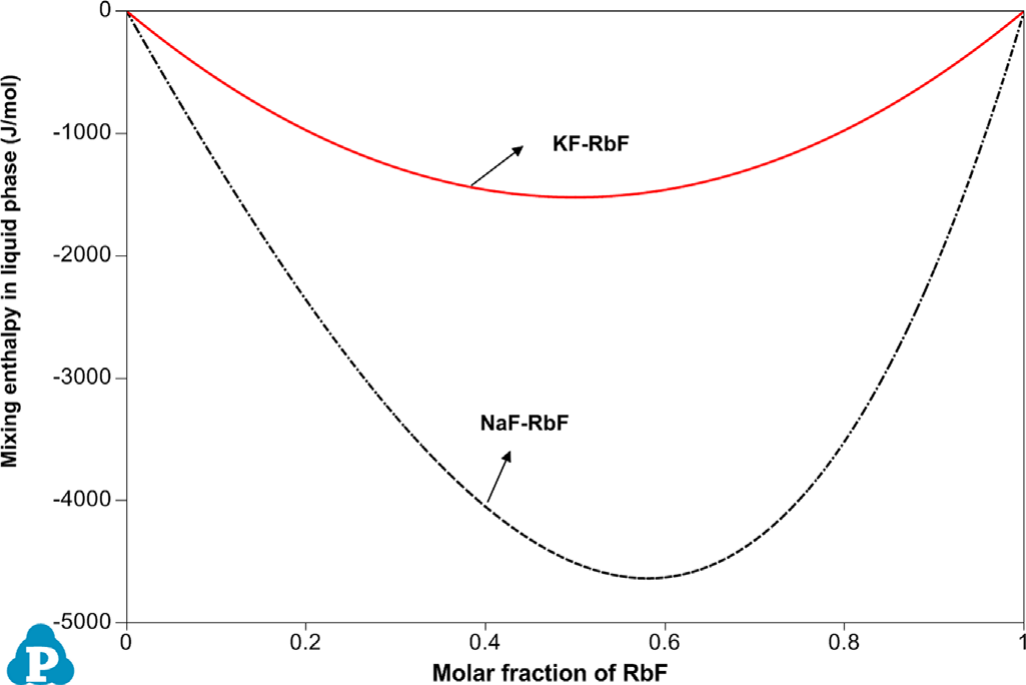
Calculated mixing enthalpy in liquid phase of AF-RbF (A=Na and K) at 1500°C.

Fig. 1, Fig. 2 demonstrate the calculated mixing enthalpy in the binary AF-ZnF_2_ (A=Na, K and Rb) and AF-RbF (A=Na and K) system, respectively, which are the classical U-shape. What׳s more, the maximum of mixing enthalpy in liquid phase is decreased with the increase of the atomic radius (atomic number). As observed from Fig. 1, Fig. 2 HmaximumNaF−ZnF2mixing−enthalpy>HmaximumKF−ZnF2mixing−enthalpy>HmaximumRbF−ZnF2mixing−enthalpy and HmaximumNaF−RbFmixing−enthalpy>HmaximumKF−RbFmixing−enthalpy that is in consistent with the generally accepted several laws.

## 2 Experimental design, materials, and methods

The data from Table 1 was cited from Factsage 6.4 database bought by ourselves. As observed in Table 2, the optimized model parameters for the NaF-KF-RbF-ZnF_2_ system are the final optimized result, by which a complete phase diagram of NaF-KF-RbF-ZnF_2_ subsystem can be plotted. Additionally, relevant thermochemistry result for NaF-KF-RbF-ZnF_2_ subsystem can also be obtained. Likewise, Fig. 1, Fig. 2 are calculated and plotted using *PanPhaseDiagram module of PANDAT software* through the optimized model parameters present in Table 2.

What’ more, the data of Table 3 are calculated through the first-principle calculation based on density function theory (DFT). First-principle calculation was applied to optimize the unit cell parameters of RbF, ZnF_2_, NaZnF_3_, RbZnF_3_, KZnF_3_ and K_2_ZnF_4_, and the optimized value and experimental lattice constants for all compounds (RbF, ZnF_2_, NaZnF_3_, RbZnF_3_, KZnF_3_ and K2ZnF_4_) are listed in Table 3. More details about the corresponding calculated method can be referred [1]. Accordingly, the relative deviation of unit cell parameters between before and after structure optimization for the NaF-KF-RbF-ZnF_2_ quaternary system is analyzed by ourselves through the function of $\frac{M_{after-optimization}-M_{before-optimization}}{M_{before-optimization}}$($M_{after-optimization}$ and $M_{before-optimization}$ stand for the unit cell parameters before and after optimization, respectively).

In addition, the comparison of calculated invariant points of the ternary system NaF-KF-ZnF_2_, NaF-RbF-ZnF_2_ and KF-RbF-ZnF_2_ from phase diagram calculation cited the developed database [1] and predicted value using the method [2] are listed in Table 5. Meanwhile, the compared data were analyzed through  by ourselves (* and ※ refer to the value from phase diagram calculation and predicted value, respectively).
