# Supplementary material for: Dataset for the thermodynamic description for the NaF-KF-RbF-ZnF2 system
Source: Data Brief. 2018 Nov 5;22:1088–93. doi: 10.1016/j.dib.2018.10.087 (PMC6376127; doi:10.1016/j.dib.2018.10.087)
Supplement: Supplementary file 1 — Supplementary material [file mmc1.docx]

**Author declaration**

The authors declared that they have no conflicts of interest to this work.

Huiqin Yin, Shuang Wu, Xueliang Wang, Long Yan, Wenguan Liu and Zhongfeng Tang
